# Immediate implantation of ultrafine fiber slow-release system based on cell electrospinning to induce osteogenesis of mesenchymal stem cells

**DOI:** 10.1093/rb/rbad113

**Published:** 2023-12-25

**Authors:** Tao Lu, Long Yang, Zhuoyang Li, Yin Liu, Shun'en Xu, Chuan Ye

**Affiliations:** Department of Orthopaedics, The Affiliated Hospital of Guizhou Medical University, Guiyang 550004, China; Department of Orthopaedics, The First People’s Hospital of Guiyang, Guiyang 550004, China; Department of Orthopaedics, The Affiliated Hospital of Guizhou Medical University, Guiyang 550004, China; Center for Tissue Engineering and Stem Cell Research, Guizhou Medical University, Guiyang 550004, China; Department of Otolaryngology Head and Neck Surgery, Xinqiao Hospital, Army Medical University (Third Military Medical University), Chongqing 400037, China; Center for Tissue Engineering and Stem Cell Research, Guizhou Medical University, Guiyang 550004, China; Department of Dental implant, Stomatological Hospital of Guiyang, Guiyang 550000, China; Department of Orthopaedics, The Affiliated Hospital of Guizhou Medical University, Guiyang 550004, China; Center for Tissue Engineering and Stem Cell Research, Guizhou Medical University, Guiyang 550004, China; Department of Orthopaedics, The Affiliated Hospital of Guizhou Medical University, Guiyang 550004, China; Center for Tissue Engineering and Stem Cell Research, Guizhou Medical University, Guiyang 550004, China

**Keywords:** bone tissue engineering, cell electrospinning, *in vivo* induction, immediate implantation, biomaterials

## Abstract

This study presents the development and evaluation of a poly(3-hydroxybutyrate-co-4-hydroxybutyrate) (P34HB) ultrafine fiber slow-release system for *in vivo* osteogenic induction of human umbilical cord mesenchymal stem cells (HUCMSCs). Utilizing dual-nozzle and cell electrospinning techniques, the system encapsulates L-ascorbic acid-2-phosphate magnesium (ASP), β-glycerophosphate sodium and dexamethasone (DEX) within the fibers, ensuring sustained osteogenic differentiation. The scaffold’s morphology, characterization, hydrophilicity, mechanical properties and cellular behavior were examined. Immediate subcutaneous implantation in rabbits was conducted to observe its ectopic osteogenic induction effect. Successfully fabricated P34HB ultrafine fiber slow-release system. Characterization confirmed the uniform distribution of HUCMSCs and inducing components within the scaffold, with no chemical reactions affecting the active components. *In vitro* tests showcased a prolonged release of DEX and ASP, while biocompatibility assays highlighted the scaffold’s suitability for cellular growth. Alizarin Red, type I collagen, and osteopontin (OPN) staining verified the scaffold’s potent osteogenic induction effect on HUCMSCs. Notably, immediate implantation into New Zealand White rabbits led to significant new bone formation within 8 weeks. These findings underscore the system’s potential for immediate *in vivo* implantation without prior *in vitro* induction, marking a promising advancement in bone tissue engineering.

## Graphical Abstract

**Graphical Abstract:**
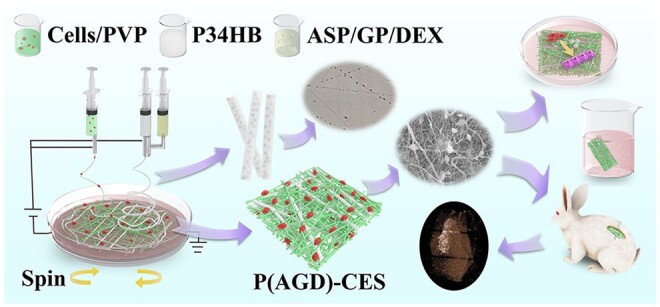

## Introduction

Bones play numerous vital roles in the human body, providing a structural framework for muscles and other tissues, facilitating movement and protecting internal organs from injury [1]. Large bone defects resulting from bone tumors, injuries and other skeletal diseases often cannot self-heal through the body’s natural repair mechanisms. Thus, bone grafts, either natural or synthetic, are required to replace the diseased or missing bone [1, 2]. Although traditional grafting techniques are effective, they may lead to surgical complications and suboptimal outcomes. This has propelled the rise of tissue engineering solutions, especially bone tissue engineering, as promising alternatives [3, 4].

In the evolution of bone tissue engineering, scaffolds constructed using biomaterials, combined with stem cells, have been osteoinduced *in vitro* for 2–3 weeks before implantation into bone defect sites in animals, achieving promising bone defect repair outcomes [5]. Electrospinning is a prevalent technique for fabricating tissue engineering scaffolds for bone tissue engineering. During electrospinning, precursor solutions transform from droplets to fine fibers under a high-voltage electric field. The collected ultrafine fibers form a fibrous membrane, which mimics the extracellular matrix, providing a conducive environment for cell proliferation and osteoinduction. Our previous work has validated this [6, 7]. Despite the significant advantages of electrospun scaffolds, they have limitations, one of which is the uneven distribution of seeded cells inside and outside the scaffold, leading to poor cell infiltration. To overcome this limitation, a novel method, cell electrospinning (CES), has been proposed. CES, a technique based on electrospinning, produces fibers that can embed live cells. The primary distinction between CES and traditional electrospinning lies in the use of live cells [8], offering immediate implantation conditions for our experiments.

In addition to fabrication techniques, biomaterials are indispensable in bone tissue engineering. The choice of biomaterial is crucial. Poly(3-hydroxybutyrate-co-4-hydroxybutyrate) (P34HB), a polyhydroxyalkanoate derivative, stands out due to its mechanical robustness, biocompatibility and biodegradability [9]. Its superior surface properties further enhance its appeal as a scaffold material [10]. Consequently, P34HB has garnered significant interest as a carrier for the sustained release of bioactive molecules or as a degradable implant material. Our preliminary investigations have confirmed its excellent biocompatibility, biodegradability, and promotive effect on the osteogenic differentiation of bone marrow mesenchymal stem cells [6, 11]. In this study, we continue to employ P34HB for the development of CES fiber scaffolds for osteogenesis.

Furthermore, ASP, β-glycerophosphate sodium (GP) and dexamethasone (DEX) are essential supplements in osteogenic cell induction culture media. *In vitro*, ascorbic acid has been proven to promote osteoblast differentiation [12]. Due to the strong reducing nature of ascorbic acid, its stability in external environments is relatively low. ASP, a long-acting derivative of ascorbic acid, is gradually replacing ascorbic acid in specific applications due to its superior stability and similar function [13, 14]. Glycerophosphate acts as a phosphate group donor in bone matrix mineralization studies [15]. DEX, a widely used synthetic glucocorticoid, promotes osteoblast differentiation [16]. However, reports on these three components inducing stem cell osteogenesis *in vivo* are scarce. Wang et al. fabricated a lysine diisocyanate osteogenic scaffold with sustained release of ascorbic acid, β-glycerophosphate and DEX, studying its *in vitro* osteogenic induction capability on stem cells [17]. Yet, the combination of these three inducing components with CES technology for immediate implantation and exploration of their *in vivo* osteogenic induction capability remains unexplored. In studies of drug-carrying electrospun fibers, a high concentration of a drug is often distributed on the fiber surface, which exhibits an initial burst release. Nanofibers prepared by core-shell nozzles can confine the drug to the core layer, which is expected to limit the drug release at the initial stage [18]. Coaxial electrostatic spinning is a common method for preparing core-shell fibers. By this technique, drugs can be encapsulated inside the fibers and the release time can be effectively prolonged as the encapsulation rate increases [19].

In this research, we introduce a novel P34HB-based fiber slow-release system, crafted using dual-nozzle CES. Combined with a core-shell nozzle, this system embeds HUCMSCs into P34HB fibers loaded with ASP, GP and DEX for immediate *in vivo* application. It consistently promotes osteogenic differentiation of HUCMSCs.

## Materials and methods

### Materials

The primary materials and equipment used are as follows: Electrospinning device (Ucalery, Beijing, China), Electron microscope (Hitachi, Japan), Universal mechanical testing machine (MTS Systems Corporation, China), Incubator (Thermo, China), Static contact angle measurement system (JC2000C, Shanghai), P34HB (300 kDa, aseptic grade, MedPHA Biotech Co., Ltd, China) and PVP (360 kDa, Sigma-Aldrich, USA). All chemicals and reagents used in this study were purchased from Sigma-Aldrich (St Louis, USA), unless otherwise specified.

### Preparation of ultrafine fiber slow-release system

Two hundred fifty milligrams ASP, 80 mg GP and 1 mg DEX were dissolved in 3 ml deionized water to obtain solution A, which was fully filtered three times using a 0.22-µm filter to remove bacteria. Solution B was obtained by dissolving P34HB in a mixture of organic solvents (chloroform and dimethylformamide, 4:1, v/v) (Sigma, St Louis, MO, USA) to configure an 8% solution of P34HB. Ten percent of PVP was configured using PBS and filtered to remove bacteria, and 1 × 10^6^/ml of HUCMSCs (ZhongGuan Biotechnology Co., Ltd, China) was mixed to obtain Solution C. The electrospinning equipment and the surrounding environment were fully sterilized with 75% alcohol and ultraviolet light before the start of the experiment. The instruments used in the experiment were autoclaved and sterilized, and the aseptic operation was strictly observed during the experiment [20]. Solutions A and B were placed in the inner and outer layers of a coaxial nozzle 1, connected to a 5-ml glass syringe. Receiving distance 10 cm, voltage 8 kV, pushing speed inner layer 0.3 mm/min, outer layer 0.03 mm/min. Receiving distance 10 cm, voltage 8 kV, pushing speed inner layer 0.3 mm/min, outer layer 0.03 mm/min. The receiver was a rotating petri dish containing culture medium, with a diameter of 15 cm and a rotation speed of 10 rpm/min. Cell/fiber scaffolds P(AGD)-CES group containing ASP, GP and DEX with a thickness of 0.1 cm and a length and width of 1 cm × 1 cm were collected and cut. P(AGD) group without CES was collected using the same conditions as well as pure P34HB (P group) fiber scaffolds for spare. P-AGD group was induced *in vitro* with the addition of osteogenic induction medium for P34HB.

### Optical microscopy, scanning electron microscopy and ultrafine fiber diameter distribution

Observation of P(AGD) group fibers and internal DEX, GP, ASP structures on glass slides. After drying, gold spraying and other treatments, SEM images were obtained using an SEM (XL30, FEI, USA) for the P(AGD)-CES group, P(AGD) group and P group. The diameter of the ultrafine fibers was calculated using ImageJ.

### Energy dispersive X-ray analysis

The chemical elements of the P(AGD) and P group fiber scaffold were analyzed using energy dispersive X-ray analysis (EDX) (Thermoscientific Apreo S, USA). EDX analyses to characterize the elemental composition were performed from the total area of each root canal sealer specimen by using EDAX Team software.

### X-ray diffraction analysis

X-ray diffraction analysis (XRD) spectra of the P(AGD)-CES (cell-free), P(AGD) and P34HB group scaffolds were recorded on an X-ray diffractometer in the 2θ range of 5°–40°. CuKα radiation (wavelength, 1.54 A°; filament current, 30 mA; voltage, 40 kV) was used to produce X-rays. The crystallinity of the samples was assessed from the XRD patterns by separating the amorphous and crystalline portions and using the following expression:

(1) Crystallinity (%)=[Acr/(Acr+Aam)]×100,

where Acr is the area under the crystalline peak and Aam is the area under the amorphous portion.

### Porosity

The porosity of P(AGD)-CES (cell-free), P(AGD) and P34HB group scaffolds was measured using a high-performance, fully automated piezomercury tester, with a pressure range of 0.5–33000 psi and pore size range of 50 μm–5 nm (AutoPore V 9600, Micromeritics, USA).

### Fourier transform infrared spectroscopy

The chemical structure of the P(AGD)-CES (cell-free), P(AGD) and P34HB group scaffolds was determined using Fourier transform infrared (FT-IR) spectroscopy using the KBr method. Infrared spectra were recorded in the energy range of 4000–500 cm^−1^, with a resolution of 4 cm^−1^. Each spectrum was an average of eight scans.

### Mechanical testing

Fiber membranes from P(AGD)-CES (cell-free), P(AGD) and P34HB group scaffolds were cut into small pieces measuring 60 mm × 15 mm, with an effective tensile length of 40 mm. Tensile tests were conducted using an electronic testing machine (100 N sensor, stretching speed of 5 mm/min). The tensile strength, elastic modulus and elongation at break were averaged for three samples, and stress–strain curves were plotted (Meters Industrial Systems, Inc., China).

### ASP, DEX slow-release curve

The slow release of ASP or DEX was determined according to the reported method [21]. The P(AGD) group bioactive scaffolds were cut and weighed, and each group was 100 mg. They were then transferred to small tubes containing 1 ml PBS. These tubes were kept at 37°C, shaking at 60 rmp/min in a shaking incubator. Every 48 h, 1 ml was taken out from each tube and replaced with fresh PBS solution. The release solutions were analyzed using a Shimadzu-2700 UV-Visible spectrophotometer, where λ_ASP_ = 260 nm and λ_DEX_ = 262 nm. Analyze and calculate both release formulas using OriginPro.

### Hydrophilicity

The hydrophilicity of the P(AGD)-CES (cell-free), P(AGD) and P34HB group scaffolds was evaluated through contact angle (water) and water absorption analyses. A drop of water was placed on the fiber membrane, and the water droplet was captured to measure the contact angle. The samples (length × width × thickness: 5 cm × 5 cm × 0.1 mm) were soaked in PBS at 37°C. After 4 h, the water absorption rate of the fiber pad was calculated using the following formula:

(2) Water absorption rate (%)=[(Mw−Md)/Md]×100%,

where Md is the original weight of the bioactive scaffold and Mw is the weight of the scaffold after soaking in PBS.

### *In vitro* degradation experiment

P(AGD)-CES (cell-free), P(AGD) and P34HB group scaffolds were cut into 1 cm × 1 cm pieces with thickness of 0.1 mm, vacuum freeze-dried for 24 h, weighed and recorded. Then, the membranes were placed in small beakers containing 10 ml of physiological saline. These beakers were kept shaking in a 37°C water bath. The degradation rate of the fiber membranes was calculated every week for 2 months.

(3) Degradation rate=[(M0−M1)/M0]×100%,

where M0 is the weight before degradation and M1 is the weight after degradation.

### CES cell survival rate, biocompatibility and HUCMSCs proliferation

The P(AGD)-CES group with a thickness of 0.1 mm and a length and width of 1 × 1 cm was co-cultured in a six-well plate. The same-sized scaffolds of the P(AGD), P-AGD and P34HB groups were inoculated with 1 × 10^6^ HUCMSCs per well for *in vitro* culture. Osteogenic induction medium was added to the P-AGD group. CES survival was calculated by assessing cell viability in the P(AGD)-CES group using a live/dead staining kit after 1 day of culture. Cell viability on the scaffolds of P(AGD)-CES group, P-AGD group, P-AGD group, P group was assessed using live/dead staining kits after 7 and 14 days and photographed using a two-photon microscope (FVMPE-RS, Olympus, Japan). HUCMSCs proliferation was analyzed using ImageJ.

### SEM of HUCMSCs adhesion

The P(AGD)-CES group with a thickness of 0.1 mm and a length and width of 1 × 1 cm was co-cultured in a six-well plate. The same-sized scaffolds of the P(AGD), P-AGD and P34HB groups inoculated with 1 × 10^6^ HUCMSCs per well for *in vitro* culture. After 7 and 14 days, the four groups of cell/scaffold composites were fixed overnight in 2.5% glutaraldehyde, dehydrated through a gradient of ethanol, dried and gold-sprayed. The distribution and morphology of cells on the fiber scaffolds were observed using an SEM at an accelerating voltage of 20 kV.

### Osteogenesis promotion of scaffolds on mesenchymal stem cells *in vitro*

The P(AGD)-CES group with a thickness of 0.1 mm and a length and width of 1 × 1 cm was co-cultured in a six-well plate. The same-sized scaffolds of the P(AGD), P-AGD and P34HB groups were inoculated with 1 × 10^6^ HUCMSCs per well for *in vitro* culture. Immunohistochemical staining was performed after 7 and 14 days using calcein, type I collagen and osteopontin staining kits to evaluate the *in vitro* osteogenic induction of HUCMSCs under the four conditions. Fluorescent photos were taken using a two-photon microscope, and analysis was performed using Image-ProPlus.

### Ectopic osteogenesis of fiber slow-release system

*In vivo* ectopic osteogenesis induction studies were conducted on New Zealand White rabbits (20 rabbits, 2–3 kg). This animal experiment was ethically reviewed and approved by the Ethics Committee of Guizhou Medical University (No. 1901083). All procedures were conducted according to our animal research institution guidelines and in accordance with the UK Animals (Scientific Procedures) Act of 1986. The study complied with the ‘Guide for the Care and Use of Laboratory Animals’ published by the US National Institutes of Health (NIH Publication No. 8023, revised 1978). P(AGD)-CES group scaffolds with a thickness of 0.1 mm and a length and width of 1 × 1 cm and P(AGD), P-AGD and P34HB group scaffolds inoculated with HUCMSCs at 1 × 10^6^/well were prepared and set aside. After anesthetizing the animals and shaving their backs, a 2-cm incision was made subcutaneously, the cell scaffold was implanted and the wound was sutured. The P(AGD)-CES group was implanted under the rabbit skin immediately after preparation; the P(AGD), P-AGD and P34HB groups were implanted under the rabbit skin after 2 weeks of co-culture *in vitro*; and the P-AGD group was added with *in vitro* osteogenic induction medium. Two months later, the newly formed tissue was removed, fixed in 4% polyformaldehyde and observed for new bone formation using micro-CT.

### Histological staining

All samples removed from the subcutis were fixed with 4% paraformaldehyde, dehydrated with gradient ethanol and embedded in paraffin. They were then cross-sectioned at 5 μm for histological analysis. Before staining, the sections were deparaffinized and rehydrated. Four groups of subcutaneous samples were stained for *in vivo* osteogenesis using HE, Alizarin Red and Masson staining kits, and the slices were viewed and photographed under a light microscope and analyzed using Image-ProPlus.

### Statistical analysis

All experimental data are presented as mean ± standard deviation. One-way analysis of variance was used, with differences considered statistically significant at *P < 0.05 or **P < 0.01 (*n* ≥ 3).

## Results

### Microscopic morphology of fiber slow-release system

**Figure 1.**
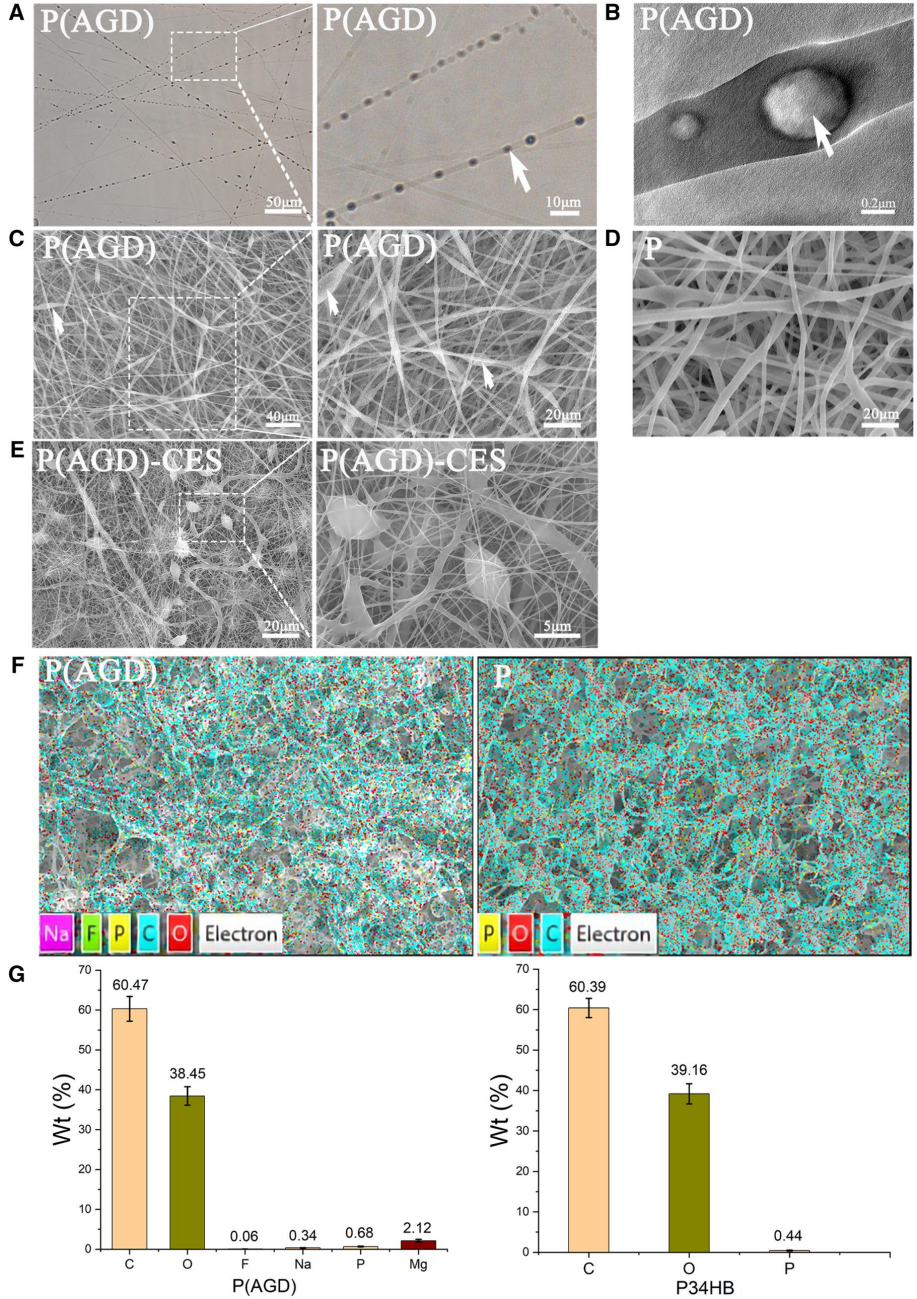
Morphological observation and energy dispersive X-ray analysis of the fiber slow-release system. Optical microscope observation of P(AGD) fibers containing ASP, GP and DEX (**A**) and transmission electron microscopy (**B**) and SEM (**C**), SEM of pure P34HB fibers (**D**), SEM of CES fiber slow-release system (**E**), EDX analysis and its elemental content of P(AGD) and P34HB (**F, G**). White arrows point to DEX, ASP and GP encapsulated in ultrafine fibers.

P(AGD)-CES group, P(AGD) group and P group fiber scaffolds were successfully prepared, and their ultrafine fiber structures were observed (Figure 1A and C–E). Under the electron microscope, the ultrafine fibers were distributed disorderly, and cells were evenly embedded within the fiber network (Figure 1E). Under the optical microscope, crystals formed by the three effective components ASP, GP and DEX were evenly distributed inside the ultrafine fibers (Figure 1A). Transmission electron microscopy revealed the crystalline structure of ASP, GP and DEX within the fibers (white arrow) (Figure 1B). The average diameter of P34HB was measured to be 1.65 ± 0.56 μm by SEM.

### Energy dispersive X-ray analysis

Through EDX, the distribution and content of the three inducers DEX, ASP and GP in the fiber membrane were clarified. P34HB contains four elements: C, H, O and P (Figure 1F). The molecular formula of DEX is (C22H29FO5), ASP is (C6H6Mg1.5O9P·×H2O), and GP is (C3H7Na2O6P·5H2O). Scanning revealed that the pure P group contains C: 60.39 ± 2.37%, O: 39.16 ± 2.51% and P: 0.44 ± 0.11%. In the P(AGD) fibers, the presence of F, Mg and Na elements confirmed the uniform distribution of DEX, ASP and GP within the ultrafine fibers (Figure 1G). The mass percentages of the three elements in the P(AGD) group were measured as F: 0.06 ± 0.01%, Na: 0.34 ± 0.05% and Mg: 2.12 ± 0.35% (Figure 1G). Thus, the mass percentages of the three substances in the prepared ultrafine fiber slow-release system were calculated as DEX: 1.24 ± 0.21%, ASP: 4.73 ± 0.78% and GP: 15.5 ± 2.28%.

### X-ray diffraction analysis

**Figure 2.**
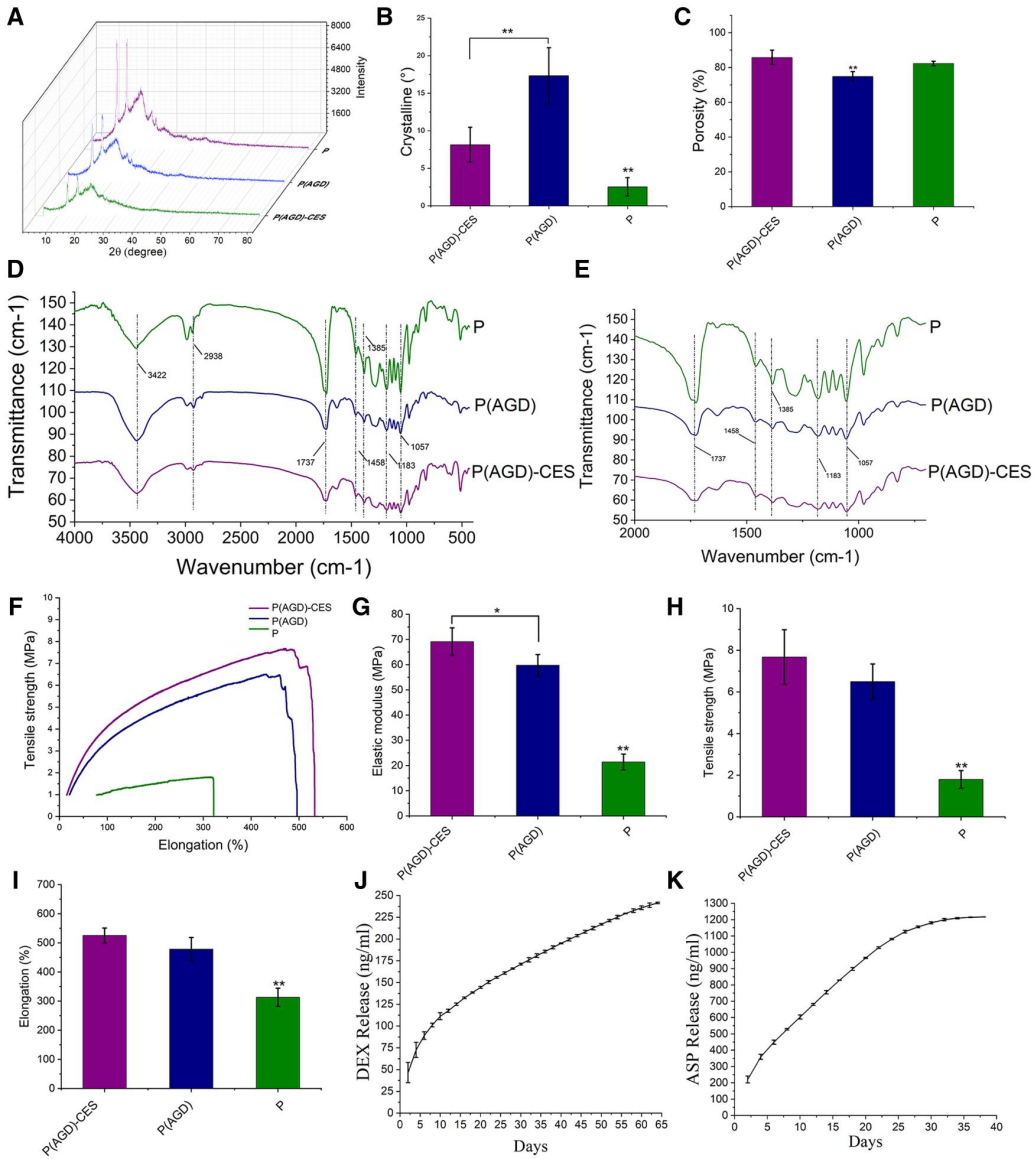
Characterization of physical and chemical properties of fiber scaffold. Where the P(AGD)-CES group did not add cell. The P(AGD)-CES group, P(AGD) group, P group fiber scaffold XRD analysis (**A**), crystallinity quantitative (**B**), porosity test (**C**), FT-IR spectrum (**D**) and local FT-IR spectrum (**E**). The mechanical properties of the P(AGD)-CES group, P(AGD) group and P group fiber scaffolds were tested. Stress–strain curve (**F**), elastic modulus (**G**), tensile strength (**H**), fracture elongation rate (**I**), cumulative release curves of DEX (**J**) and ASP (**K**) in the P(AGD)-CES group. *P < 0.1, **P < 0.01.

The XRD analysis spectra of the P(AGD)-CES, P(AGD) and P34HB group fiber scaffolds were tested (Figure 2A). The crystallinity of each group was calculated as P(AGD)-CES group: 8.13° ± 2.12°, P(AGD) group: 17.32° ± 3.74° and P34HB: 2.52° ± 1.23° (Figure 2B). It was concluded that after adding DEX, ASP and GP to P34HB, the crystallinity of the fiber slow-release system significantly increased. The increase in crystallinity led to an increase in tensile strength, which was reflected in the mechanical property testing.

### Porosity

The average porosity of the scaffolds was determined by the pressed mercury porosimetry method (Figure 2C), which reflects the environment for cell growth in the scaffolds and the ability of cells to exchange nutrients with the outside world. Scaffolds with high porosity are more favorable for providing a microenvironment for cell growth. Porosity was measured as 82.3° ± 1.3° for the P34HB group, 74.9° ± 2.7° for the P(AGD) group and 85.81° ± 4.1° for the P(AGD)-CES group. From the above results, we can conclude that all three groups of scaffolds have high porosity. The addition of DEX, ASP and GP to the P34HB fibers decreased the porosity, but the porosity was enhanced after the addition of CES. This is closely related to the addition of PVP, which releases more space after hydrolysis. The increase in porosity of the P(AGD)-CES group provides a better microenvironment for the growth of HUCMSCs.

### FT-IR spectroscopy

The FT-IR of the P(AGD)-CES, P(AGD) and P34HB group fiber membranes are shown in Figure 2D. The wavenumbers of the P34HB fiber membrane are 2985, 2938 cm^−1^ peaks for the C–H stretching vibration of the carbon–hydrogen bond, the 1737 cm^−1^ absorption peak corresponds to the stretching vibration of the carbonyl C=O, the 1458 and 1385 cm^−1^ absorption peaks are for the bending vibration of the carbon–hydrogen bond C–H, the 1183 cm^−1^ absorption peak corresponds to the stretching vibration of C–O–C and the 1057 cm^−1^ absorption peak is for the coupled vibration of the C–O–C–O–C bond (Figure 2E). From this result, it can be analyzed that in the P(AGD)-CES and P(AGD) groups with added PVP and ASP, GP and DEX, only the intensity of each peak is weakened, and no new peaks are found. No new groups are formed, indicating that no chemical reactions were found between the various substances.

### Mechanical testing

The mechanical properties of the slow-release fiber scaffold were evaluated, and the corresponding stress–strain curve was plotted (Figure 2F). The elastic modulus of the fiber membrane in P34HB was 21.42 ± 3.1 MPa, P(AGD) was 59.78 ± 4.2 MPa and P(AGD)-CES was 69.15 ± 3.1 MPa (Figure 2G). The tensile strength of the fiber membrane in P34HB was 1.8 ± 0.4 MPa, P(AGD) was 6.5 ± 0.8 MPa and P(AGD)-CES was 7.68 ± 1.31 MPa (Figure 2H). The fracture elongation rate of the fiber membrane in P34HB was 313.28 ± 31.12%, P(AGD) was 478.35 ± 40.26% and P(AGD)-CES was 525.6 ± 25.45% (Figure 2I). It was concluded that after adding DEX, ASP and GP to the fiber membrane, the elastic modulus and tensile strength of the fiber membrane were enhanced to a certain extent. After adding CES, the elastic modulus and tensile strength were further increased, which is related to the addition of PVP. Good mechanical properties are essential for bone tissue engineering scaffolds, and DEX, ASP, GP and PVP were added to enhance the mechanical properties of fiber scaffolds, which is beneficial for tissue-engineered bone.

### DEX and ASP slow-release curve

The slow release of ASP and DEX from P(AGD) group fibers was tested by UV spectrophotometry, and their cumulative release curves were plotted. DEX’s sustained release can last for 2 months (Figure 2J), with a larger release in the first 10 days, and a stable release of about 5–10 ng/ml/2 days after 10 days. ASP’s release can last for about 1 month (Figure 2K). This is related to the water solubility of DEX and ASP. ASP is more water soluble, while DEX dissolves slowly. Although ASP’s slow release can only last for about 1 month, since *in vitro* osteogenic induction usually only requires 14–21 days, it is sufficient to complete the osteogenic induction effect on HUCMSCs. Based on the release curves of both, we calculated the cumulative release formulas for DEX, ASP as:

Y_DEX_ = 96.87 + 1.71X; Y_ASP_ = 532.01 + 18.38X, where Y is the cumulative release concentration (measuring unit: ng/ml) and X is the number of days of release.

### Hydrophilicity

**Figure 3.**
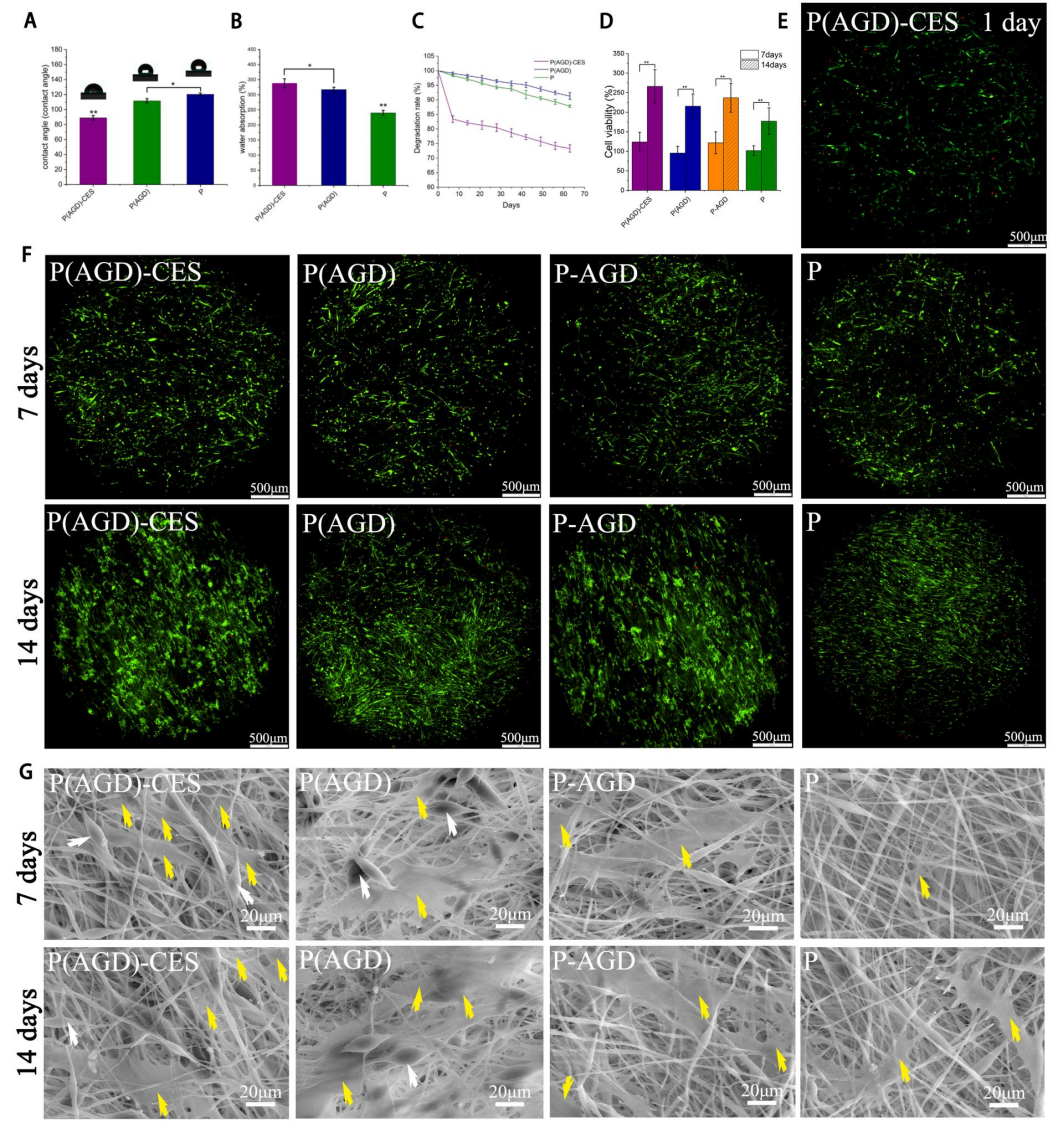
Fiber slow-release system’s hydrophilicity test, *in vitro* degradation, biocompatibility and proliferation adhesion electron microscopy observation. Contact angles of the slow-release system at 0, 15 and 30 s (**A**), swelling rate (**B**), *in vitro* degradation curve (**C**), cell survival on day 1 of CES (**E**), cells/scaffolds were cultured *in vitro* for 7 and 14 days for cell proliferation (**D**), live/dead staining imaging for 7 and 14 days (**F**), SEM of HUCMSCs on the bioactive fiber membrane on the 7 and 14 days (**G**). White arrows point to DEX, ASP and GP encapsulated in ultrafine fibers, and yellow arrows point to the stem cells (scale: 500 μm, 20 μm). *P < 0.1, **P < 0.01.

The hydrophilicity and water absorption capacity of the bioactive fiber membrane were tested. The water contact angles of the fiber membranes were P34HB (120.34° ± 1.83°), P(AGD) (111.63° ± 3.02°) and P(AGD)-CES (88.97° ± 3.05°) (Figure 3A). With the addition of DEX, ASP and GP, the hydrophilicity of the fiber membrane was enhanced. The inclusion of PVP can significantly improve the hydrophilicity of the bio-fiber membrane, with the P(AGD)-CES group having the best hydrophilicity. The water storage capacity of the three groups of fiber membranes soaked in PBS for 4 h was also tested. The P(AGD)-CES group had the strongest water storage capacity, reaching (338.1 ± 15.13%), followed by the P(AGD) group (317.3 ± 8.08%), while the P34HB group had a poorer water storage capacity of (240.1 ± 8.47%) (Figure 3B). Hydrophilic materials are friendly to cell adhesion and proliferation, and they can store more water and nutrients for better nourishment of cells [7]. P(AGD)-CES group has the best hydrophilicity and water storage capacity, and it is more suitable to be used for bone tissue engineering scaffold preparation compared to other groups.

### *In vitro* degradation

*In vitro* degradation of the fiber scaffold membrane was verified (Figure 3C). The P34HB fiber membrane decreased 6.2 ± 0.72% in the first month and 12.2 ± 0.41% in the second month in mass. The P(AGD) group decreased 4.3 ± 0.77% in the first month and 8.7 ± 1.11% in the second month. The P(AGD)-CES group decreased 21.3 ± 1.42% in the first month and 26.7 ± 1.28% in the second month. Since PVP is a highly hydrophilic biomaterial, the biodegradability of the P(AGD)-CES group containing PVP is better than other groups, which can degrade quickly and release space for cell growth, promoting cell growth and development.

### CES cell survival rate, biocompatibility and HUCMSCs proliferation

The cell survival rate of CES was observed by 1-day live/dead staining imaging (Figure 3E). The survival rate was calculated to be 88 ± 4.3% by the number of live/dead cells at an electric field of 0.08 kV/mm. This indicated that the safety of HUCMSCs in CES could be ensured with less damage by voltage during electrospinning. Good biocompatibility of the fibrous scaffolds was observed by live/dead staining imaging after 7 and 14 days of co-culture of the fibrous scaffolds with HUCMSCs in the P(AGD)-CES, P(AGD) group, P-AGD, and P34HB groups (Figure 3F). The cells were well grown and a large number of green live cells and a small number of red dead cells were observed. This proves that P34HB and its internal ASP, GP and DEX have less toxic effects on cell growth and reproduction, and the materials we used have good biocompatibility. Cell proliferation rates at 7 and 14 days were calculated by ImageJ (Figure 3D), 124.12 ± 24.54% at 7 days and 266.18 ± 42.87% at 14 days in the P(AGD)-CES group, 95.72 ± 17.27% at 7 days and 215.78 ± 31.02% at 14 days in the P(AGD) group, 121.96 ± 28.18% at 7 days and 237.16 ± 36.77% at 14 days in the P-AGD group, 101.96 ± 12.36% at 7 days and 177.27 ± 34.03% at 14 days in the P group. Comparison of 7-day HUCMSCs showed nearly 1-fold cell expansion at 14 days, with no statistical difference in cell proliferation between groups.

### SEM of HUCMSCs adhesion

Cell scaffolds co-cultured for 7 and 14 days were scanned with SEM to observe the behavior of the cells (Figure 3G). In SEM, it can be observed that the cells of P(AGD)-CES group are uniformly distributed between the fibers, the cells are in contact with each other, and they grow in three-dimensional space within the scaffold with good morphology. The pseudopods and secreted extracellular matrix of the cells could be seen, and calcium salt (CS) deposition and scaffold mineralization could be observed. The cells in the P(AGD), P-AGD and P34HB groups all showed two-dimensional growth only on the surface of the scaffold. Their pseudopods could not extend into the scaffold, and their cellular activities and behaviors would be spatially limited.

### Scaffold promotes osteogenic differentiation of HUCMSCs *in vitro*

**Figure 4.**
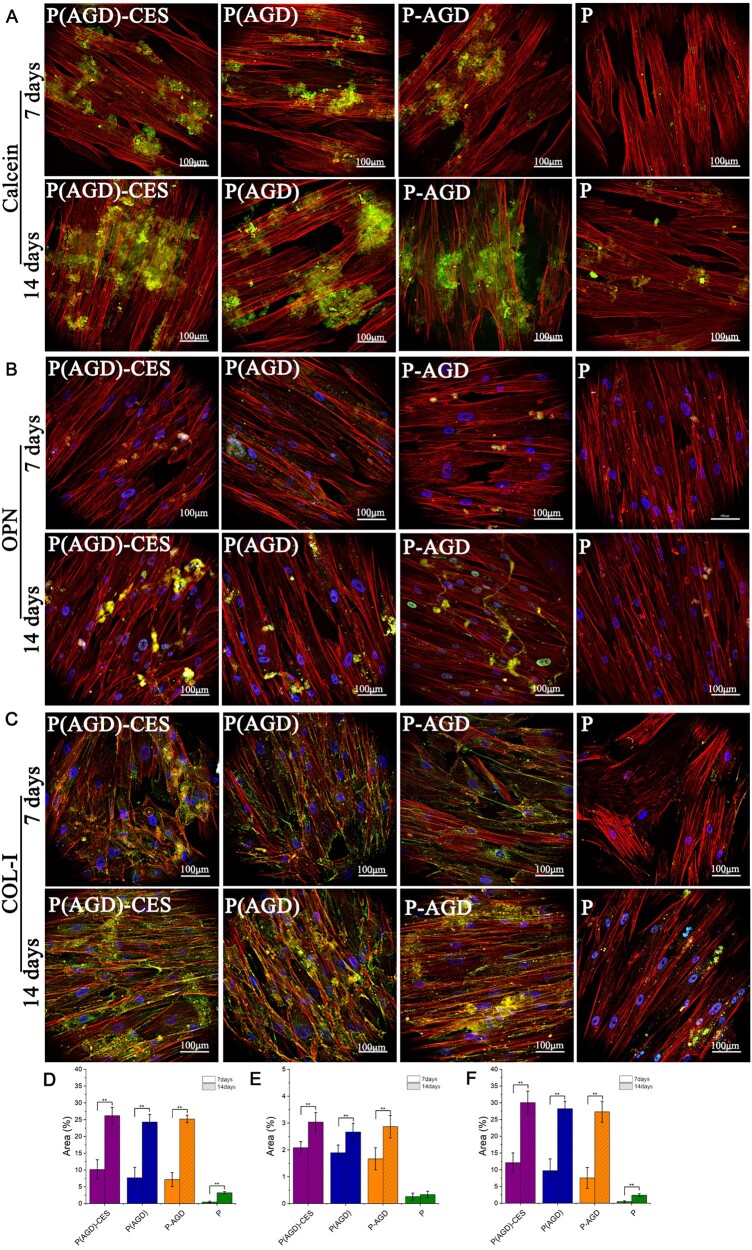
Calcein (**A**), osteopontin (**B**) and type I collagen (**C**) staining of cells/scaffold co-cultured for 7 and 14 days in the P(AGD)-CES group, P(AGD) group, P-AGD group and P group. Calcein (**D**), osteopontin (**E**) and type I collagen (**F**) staining positive area quantification for 7 and 14 days. Where red represents the cell skeleton, blue represents the cell nucleus and the yellow-green staining represents calcium nodules/osteopontin/type I collagen (scale: 100 μm). **P < 0.01.

The effect of osteogenic differentiation *in vitro* can be indirectly reflected by the staining of calcein, type I collagen and osteopontin. After 7 and 14 days of cell/scaffold co-culture, the osteogenic differentiation effect of HUCMSCs *in vitro* was observed using calcein (Figure 4A), osteopontin (Figure 4B) and type I collagen staining kits (Figure 4C). Using two-photon microscopy to take photographs, the software ImageJ measured the content of calreticulin, type I collagen, and osteoblastin in each group. Quantification of positive areas in calcein staining was 10.16 ± 2.9% at 7 days and 26.20 ± 2.46% at 14 days in the P(AGD)-CES group, 7.70 ± 3.1% at 7 days and 24.34 ± 2.3% at 14 days in the P(AGD) group, 7.16 ± 2.1% at 7 days and 25.22 ± 1.1% at 14 days in the P-AGD group, 0.5 ± 0.2% at 7 days and 3.25 ± 0.3% at 14 days in the P34HB group (Figure 4D). Quantification of positive areas in osteopontin staining was 2.08 ± 0.23% at 7 days and 3.04 ± 0.36% at 14 days in the P(AGD)-CES group, 1.89 ± 0.29% at 7 days and 2.67 ± 0.33% at 14 days in the P(AGD) group, 1.67 ± 0.41% at 7 days and 2.87 ± 0.42% at 14 days in the P-AGD group, 0.26 ± 0.13% at 7 days and 0.34 ± 0.12% at 14 days in the P34HB group (Figure 4E). Quantification of positive areas in type I collagen staining was 12.15 ± 2.9% at 7 days and 30.06 ± 3.46% at 14 days in the P(AGD)-CES group, 9.75 ± 3.5% at 7 days and 28.26 ± 2.25% at 14 days in the P(AGD) group, 7.56 ± 3.1% at 7 days and 27.33 ± 3.12% at 14 days in the P-AGD group, 0.5 ± 0.23% at 7 days and 2.42 ± 0.42% at 14 days in the P34HB group (Figure 4F). The experimental results showed that the P(AGD)-CES group and the P(AGD) group could still induce osteogenic differentiation of HUCMSCs without the addition of osteogenic induction medium. Its induction effect was close to that of the P(AGD) group, and the difference was not statistically significant.

### Scaffold promotes HUCMSCs ectopic osteogenesis *in vivo*

**Figure 5.**
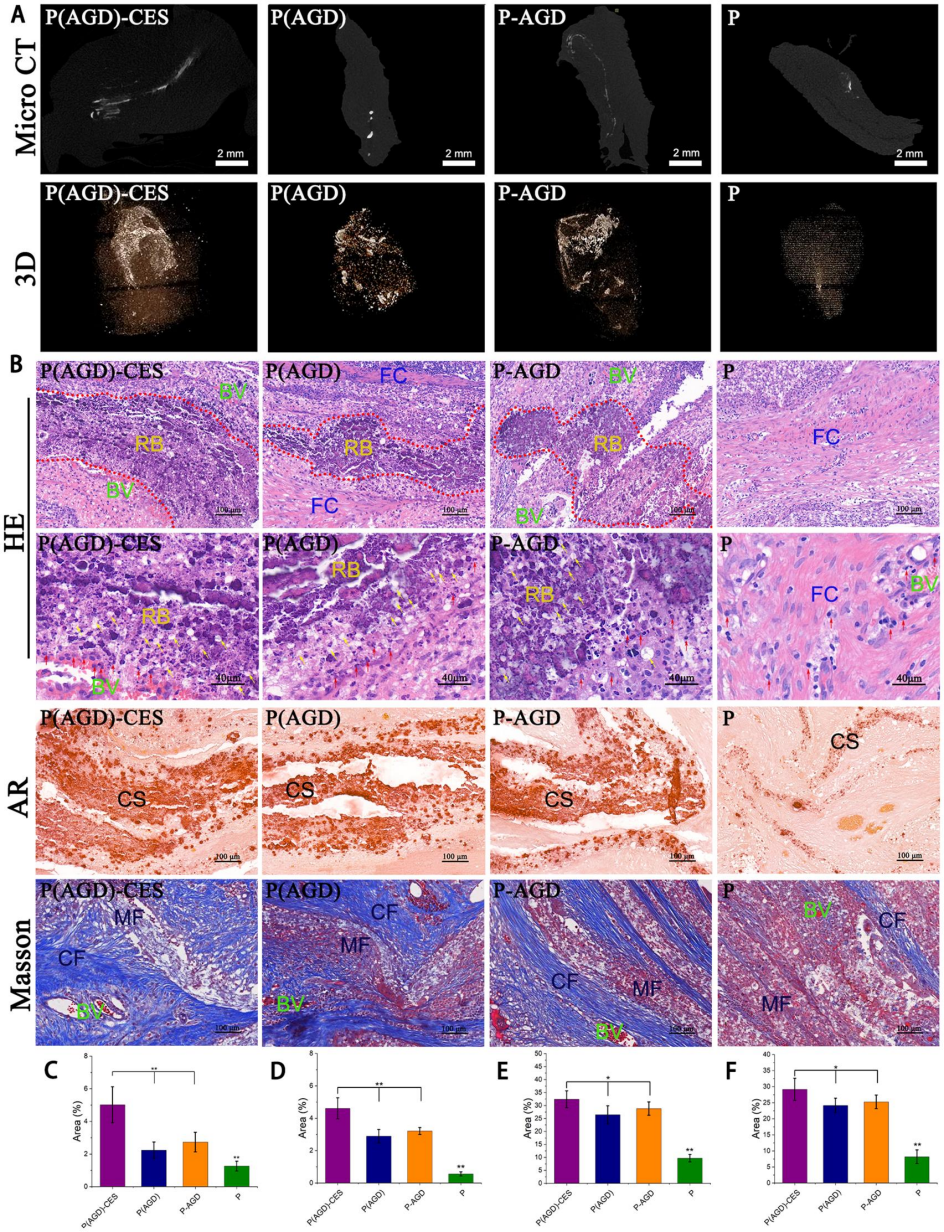
Fiber slow-release system *in vivo* ectopic osteogenesis micro-CT scan and three-dimensional reconstruction (**A**); histological staining, HE staining, Alizarin Red staining and Masson staining of the four scaffold groups (**B**); quantification of new bone in micro-CT (**C**); HE staining showed positive area (**D**); Alizarin Red staining showed positive area (**E**); and Masson staining showed positive area (**F**). Yellow arrows represent bone lacunae and osteocytes and red arrows represent osteoblasts. RB, regenerated bone; FC, fibrous connective tissue; BV, blood vessels; CS, calcium salt regions; CF, collagen fibers; MF, muscle fibers. Scale = 2 mm, 40 μm, 100 μm. *P < 0.1, **P < 0.01.

*In vivo* heterotopic osteogenesis was further evaluated for the scaffold promotion of osteogenic differentiation of HUCMSCs. The scaffolds in the P(AGD)-CES, P-AGD, P(AGD) and P groups were implanted subcutaneously in New Zealand White rabbits for 8 weeks and then removed. The newborn bone tissue inside each group was observed using micro-CT and its three-dimensional structure was simulated (Figure 5A). Newborn bone volume was measured in each group using MIMICS (Figure 5C). The P(AGD)-CES group had the largest bone volume of 5.02 ± 1.1% of the total volume. The amount of new bone in the P-AGD group of 2.24 ± 0.5% and in the P(AGD) group of 2.73 ± 0.6% was close to each other, and the difference was not statistically significant. The above results indicate that compared with *in vitro* osteogenic induction, P(AGD)-CES *in vivo* osteogenic induction was able to exert more advantages with a higher amount of new bone than other groups. HUCMSCs grown in three dimensions within the scaffold were able to undergo better osteogenic differentiation and promote more new bone generation.

### Histological staining

HE, Alizarin Red and Masson staining were used to evaluate the ability of the bioactive scaffold to form new bone (Figure 5B). HE staining confirmed the formation of new bone within the newly formed tissue block, where the ‘osteoid trabeculae’ structure is shown inside the red dashed line (regenerated bone). This can be observed in the P(AGD)-CES group, P-AGD group and P(AGD) group. Additionally, osteocytic lacunae and osteocytes (indicated by yellow arrows) and osteoblasts (indicated by red arrows) can be observed within the stained osteoid trabeculae structure in the other three groups. This demonstrates the excellent osteogenic capability of the fiber slow-release system we prepared. Alizarin Red staining further clarified the CS content in the tissue, with the deep red staining indicating the CS area. Masson staining highlighted the blue collagen fibers (CF) and red muscle fibers (MF) distributed within the newly formed tissue block, with blue collagen occupying the majority of the field of view. In addition, abundant fibrous connective tissue, blood vessels, etc. can be observed in the stained image. Based on the staining results, we concluded that the fiber slow-release system P(AGD)-CES group we prepared has excellent bone induction effects, superior to the traditional bone induction techniques of the P-AGD group and the cell electrospinning P(AGD) group. The osteogenic effects of the P-AGD group and the P(AGD) group are similar (Figure 5D–F). Additionally, a small number of osteoblasts can be observed in the control P group with HE staining, and Alizarin Red and Masson staining show a certain amount of CS and CF, which also proves that P34HB itself has a certain bone induction capability.

## Discussion

Stem cell-based bone tissue engineering has emerged as a promising approach for addressing bone defects [22, 23]. While P34HB has shown potential as a scaffold material, its inability to provide an intrinsic osteogenic environment *in vivo* necessitates external osteogenic factors. Common osteogenic inducers, including ascorbic acid, β-glycerophosphate and DEX, have been widely used *in vitro*. Studies have shown that *in vitro* systems, the induction of type I collagen, osteocalcin, bone sialoprotein and alkaline phosphatase mRNA levels by ascorbic acid, β-glycerophosphate and DEX are related to the occurrence of bone nodules [24]. Among them, ascorbic acid is an effective collagenase inhibitor that promotes collagen synthesis [25], activates alkaline phosphatase [26], induces osteoblast differentiation and stimulates cell proliferation [27]. β-glycerophosphate is a simple phosphate donor and a classic serine-threonine phosphatase inhibitor used for kinase reaction buffers [28]. DEX is a parathyroid hormone that can significantly induce osteoblast differentiation [29]. Studies have found that ascorbic acid and β-glycerophosphate promote matrix mineralization by inducing an increase in matrix vesicle neutral metalloproteinases, which is beneficial for mineral precipitation [29].

But their combined potential in an *in vivo* setting, especially when integrated with CES, remains largely uncharted. In this study, we successfully prepared a P34HB ultrafine fiber slow-release system containing three components: ASP, GP and DEX. This system can induce stem cell osteogenic differentiation by slowly releasing ASP, GP and DEX, omitting the step of adding osteogenic induction medium for induction differentiation in the experiment. We observed through a microscope and SEM that the three effective components ASP, GP and DEX were effectively wrapped inside the P34HB fiber filament. Transmission electron microscopy confirmed the crystalline structure formed by ASP, GP and DEX inside the fiber filament. This structure can achieve slow release. *In vitro* slow-release tests showed that ASP can be continuously released for 1 month, and DEX can be continuously released for 2 months. However, *in vitro* osteogenic induction culture only requires 2–3 weeks. Its mechanical properties, hydrophilicity and biodegradability also performed well in the experiment. FT-IR spectroscopy confirmed that the components ASP, GP and DEX did not undergo chemical reactions, and the effective components were retained. EDX specified the mass fraction of effective components, which is roughly the same as the required ratio of the three in the *in vitro* osteogenic induction culture medium.

Furthermore, we successfully implemented the immediate *in vivo* implantation of the P34HB ultrafine fiber slow-release system through the dual-nozzle and CES technology to perform *in vivo* induction. The addition of CES solved the problem of uneven distribution of cells inside and outside the scaffold after traditional cell seeding and poor cell infiltration. Secondly, it realized the immediate implantation of the tissue engineering scaffold into the animal body for *in vivo* self-induction osteogenesis after the preparation of the tissue engineering scaffold. This saves the *in vitro* cell induction step and can avoid uncontrollable risks such as cell aging and contamination that may be caused by *in vitro* culture, which has not been reported in previous studies. It has been shown that the electric field is an important variable affecting cell viability, and that high cell viability (90%) can be achieved with an electric field in the range of 0.05–0.075 -kV/mm [30] or a low electric field of 0.1 kV/mm [31]. In this study, cell survival was maintained at 88 ± 4.3% at an electric field of 0.08 kV/mm. The cell fiber scaffold prepared can be observed under an electron microscope and a microscope that the cell/fiber distribution is uniform. *In vitro*, it was verified that the cells have good biocompatibility, and the osteogenic staining through *in vitro* induction confirmed that its osteogenic ability is higher than the control group. After immediate implantation *in vivo* for 8 weeks, a large amount of new bone formation was observed inside the P(AGD)-CES group scaffold through micro-CT and histological staining scans. Both *in vitro* and *in vivo* experiments showed that the P(AGD)-CES fiber slow-release system we prepared has better physicochemical properties and superior osteogenic induction effects, which is an important basis for its better realization of bone tissue regeneration.

Previous studies have reported some material-based slow-release scaffolds, such as those based on PLGA, which can release DEX and ascorbic acid-2-phosphate for *in vitro* and *in vivo* osteogenic differentiation and osteogenesis [17]; scaffolds loaded with DEX-containing polylactic acid (PLLA) and chitosan (CS) mesoporous silica nanoparticles, slow-release DEX induces osteogenesis, etc. [32], all of which have shown great potential in inducing stem cell osteogenesis. Studies in tissue engineering regarding CES, such as combining 3D printing and CES, have built 3D structures with high mechanical strength for bone regeneration [31], preparation of cardiac patches for improved cardiac function by CES [33], portable handheld CES for wound repair in rats [34], etc. Compared with the scaffolds reported in the literature, the P(AGD)-CES fiber slow-release system we prepared still has these advantages, such as excellent biocompatibility and long-lasting slow-release performance, effectively promoting HUCMSCs osteogenic differentiation and CS deposition. Secondly, the slow-release system combined with CES enables immediate implantation of scaffolds into animals for *in vivo* self-induced osteogenesis, which is very novel compared with previous research. Its biomimetic structure, mechanical properties, biocompatibility, slow-release ability and excellent bone regeneration ability make it a bone regeneration material with broad application prospects. While our findings are promising, further research is imperative to elucidate the *in situ* bone regeneration and repair capabilities of our system. As the field of bone tissue engineering continues to evolve, innovations like ours pave the way for more efficient and effective therapeutic solutions.

## Conclusion

In this study, we successfully developed the P(AGD)-CES ultrafine fiber slow-release system using dual-nozzle and CES, enabling immediate *in vivo* implantation without prior *in vitro* induction. The system’s potential in promoting osteogenic differentiation of HUCMSCs, as evidenced by both *in vitro* and *in vivo* experiments, underscores its promise as a novel therapeutic tool. Given its biomimetic attributes, mechanical robustness, biocompatibility and sustained release capabilities, the P(AGD)-CES system offers an innovative approach to addressing bone defects in clinical settings. The shortcomings of this study are the lack of *in vivo* analysis of osteogenic markers and the assessment of *in situ* osteogenesis. It will be further improved in subsequent studies.
